# Comparison of Assessment by a Virtual Patient and by Clinician-Educators of Medical Students' History-Taking Skills: Exploratory Descriptive Study

**DOI:** 10.2196/14428

**Published:** 2020-03-12

**Authors:** Jean Setrakian, Geneviève Gauthier, Linda Bergeron, Martine Chamberland, Christina St-Onge

**Affiliations:** 1 Faculté de médecine et des sciences de la santé Université de Sherbrooke Sherbrooke, QC Canada

**Keywords:** virtual patients, medical history taking, automated scoring, simulation training, medical education, medical students, educational assessment, computer software, medical history–taking skills, medical history–taking skills assessment

## Abstract

**Background:**

A virtual patient (VP) can be a useful tool to foster the development of medical history–taking skills without the inherent constraints of the bedside setting. Although VPs hold the promise of contributing to the development of students’ skills, documenting and assessing skills acquired through a VP is a challenge.

**Objective:**

We propose a framework for the automated assessment of medical history taking within a VP software and then test this framework by comparing VP scores with the judgment of 10 clinician-educators (CEs).

**Methods:**

We built upon 4 domains of medical history taking to be assessed (breadth, depth, logical sequence, and interviewing technique), adapting these to be implemented into a specific VP environment. A total of 10 CEs watched the screen recordings of 3 students to assess their performance first globally and then for each of the 4 domains.

**Results:**

The scores provided by the VPs were slightly higher but comparable with those given by the CEs for global performance and for depth, logical sequence, and interviewing technique. For breadth, the VP scores were higher for 2 of the 3 students compared with the CE scores.

**Conclusions:**

Findings suggest that the VP assessment gives results akin to those that would be generated by CEs. Developing a model for what constitutes good history-taking performance in specific contexts may provide insights into how CEs generally think about assessment.

## Introduction

### Background

Virtual patients (VPs) are increasingly used in health professions education (HPE) [1,2], including the teaching of diagnostic reasoning and interviewing [3]. Despite VPs’ positive impact on learning and skill development [4-7], their usefulness and effectiveness as learning tools have been challenged [8,9], and questions have been raised about which competencies students develop through VPs [10] and how VPs align with, and complement, learning outcomes in HPE curricula [1,11]. One main issue is the lack of outcome measures to monitor the impact of VPs on student learning.

Developing and measuring specific learning outcomes is challenging for many reasons, including the inherent variability in the ways to solve complex problems in HPE [12] as well as the impact of developmental and contextual perspectives on skills and competencies [13,14]. Consequently, current outcome measures for VPs have mostly focused on pre-post satisfaction, knowledge, or global correlation with other measures or tests [15], which only provide partial insight into competency development and mastery. More specific and accurate outcome measures are required to explore further and document a VP’s potential positive impact on students’ learning. One such outcome measure is how well the assessment *by a VP* can reproduce teachers’ assessment of students’ performance.

Assessment aligned with teachers’ judgment could become an integral part of the utilization of VP software (1) by learners for individual practice with feedback by the VP on performance and (2) by teachers as a tool for illustration and evaluation. A VP could be used for assessing reasoning and interviewing skills [16-19] and would be readily acceptable to students [20]. Creating a realistic, credible, and multidimensional VP is challenging [21]. The complexity of assessing integration of reasoning and interviewing skills [22,23] adds further to the challenge.

Our goal was to develop, and implement in a VP, an automated assessment of medical students’ history-taking skills and document how this assessment aligns with the perspectives of clinician-educators (CEs).

### Assessing Medical History–Taking Skills

Medical history is central to making a correct diagnosis, with real as well as simulated patients [24,25]. Good history-taking requires both skillful diagnostic reasoning and interviewing [26]. Observation by CEs of students obtaining a patient’s history at the bedside provides a valuable (and often the only) opportunity for teaching and assessing how these twin skills are integrated [27,28]. Several tools exist and can be used to document parts of the medical history–taking skills such as the Cambridge-Calgary model [29], the History-Taking Rating Scale (HTRS) [30], the Maastricht History-taking and Advice Checklist (MAAS) [31], and the Brown Interviewing Checklist (BIC) [32].

The items assessed in these tools are broad and require human judgment to assess. For example, “picking up clues” is an item included in the section on “gathering information” of the Cambridge-Calgary model. Such items as are covered under “gathering information” may be self-evident for CEs, yet translating them into an automated assessment is complex. For instance, VP software can be set up to assess whether a student picks up verbal clues. To do this, the VP must first be programmed with specific instances in the simulation during which the patient gives a verbal clue that must be picked up. The software can then document the student’s *behavior* (did he or she act on the clue?) and use it as *evidence* that he or she did indeed pick up the clue. Picking up verbal clues is one of the many skills that could be programmed in this fashion (ie, instances and assessment of behavior), with this degree of fine granularity into a history-taking VP.

A framework is required in the development of automated assessment by VPs, modeled on how CEs’ assess history taking. Then from this framework, specific implementation rules can be programmed into a VP to provide feedback on performance to the learner (formative assessment). Once established, such a framework could eventually lead to the development of additional evaluation tools (summative assessment).

We developed a framework to precisely articulate skills assessed in history taking by breaking down their broad components into operational objective measures. To explore whether such measures can be used in the ways outlined earlier, we tested whether they were comparable with CEs’ assessments. Articulating how we assess these skills furthers our knowledge of how we assess history taking at the bedside through tools such as the Calgary-Cambridge model.

The objectives of our study were as follows: (1) to present a framework for assessing medical history–taking skills through VP software and (2) to examine, using this framework, the alignment of VP assessment with that of CEs.

### A Framework for Virtual Patient Assessment of Medical History–Taking Skills

Our goal was first to clarify expectations and assumptions about medical history–taking skills, exploring ranges of acceptable performance in the context of medical history taking [33]. Our work thus began by operationalizing expected medical history–taking skills at the clerkship level by identifying the characteristics of a successful performance.

Building on years of experience assessing the bedside skills of students such as those described by the HTRS, the MAAS, the BIC, and the Calgary-Cambridge model and through iterative consultations with colleagues from a Canadian University, the principal investigator (JS) set out to break down the skills into a framework comprising bite-sized specific instances and behaviors that can be automated and thus programmed into a VP. These were classified into 4 domains: breadth of data gathering, depth of data gathering, logical sequence of questions, and interviewing technique. These domains were then adapted to be implemented into a specific VP environment. See Table 1 for the framework’s definitions and operationalization for implementation rules.

**Table 1:** Framework for virtual patient assessment of medical history–taking skills.

Domain		Description	Implementation rules
**Breadth**			
	Breadth of data gathering	Extent of exploration to find all relevant problem areas in the patient’s situation	Symptoms identified: as percentage out of total number of relevant symptoms
**Depth**			
	Depth of data gathering	Extent of exploration to find all relevant details about each problem area	Details asked about the symptoms: percentage out of total number of details programmed in the VP^a^
**Logical sequence**			
	Sequence of questions	Logical sequence that reflects thinking through the relevant diagnostic possibilities	Differential scoring for overall order of identification of symptoms and for alternative sequences (see Multimedia Appendix 1)
**Interviewing technique**			
	Component (a): appropriate use of generic questions	Asking for generic details that apply to each and every symptom, such as duration, severity, course, and precipitating factors	Generic questions: percentage out of total questions–>scoring performances using a range established by the CE^b^
	Component (b): appropriate use of transitioning statements	Appropriate use of transitioning statements such as “yes,” “no,” “let me ask you a few questions,” and “that’s normal”. The ideal number varies from encounter to encounter	Opening and follow-up questions, interruptions, yes or no answers, reassurance and transition statements; –>scoring performances using a range established by the CE
	Component (c): appropriate flow	Avoidance of jumping from 1 topic to the next without apparent reason, or of leaving some areas not fully explored before moving on to others	Number of times the student passes from 1 category of questions (eg, GI) to another (eg cardiac)–>scoring according to acceptable numbers established by the CE
	Component (d): successful handling of KIE^c^	Combination of a number of events or instances that require an understanding of implicit communication rules (clues, misunderstandings, tangential answers, incomplete answer, vague answer, imprecise answer)	Binary scoring of success or failure of events if encountered in any given KIE

^a^VP: virtual patient.

^b^CE: clinician-educator.

^c^KIE: key interview element.

The first 2 domains (ie, breadth and depth) concerned completeness of data gathering. Are all the patient’s symptoms obtained, and are they obtained in sufficient detail? During bedside teaching, although CEs are unaware of all the patient’s symptoms and the details thereof, they routinely make a judgment of a student’s thoroughness. For the VP, we defined breadth as the percentage of the VP’s symptoms (eg, dizziness, pallor, fatigue, hematochezia) identified by the students and depth as the percentage of programmed symptom details identified (eg, dizziness for 3 weeks, worse upon standing, first instance, without loss of consciousness).

The third domain, logical sequence of questions, reflected CEs’ judgment of students navigating through a differential diagnosis. Although diagnostic reasoning cannot be assessed directly, inferences are made about students’ reasoning through the sequence of questioning about symptoms. For example, asking about fever right after finding out about a cough is taken as indirect evidence that the student entertained the possibility of an infectious cause for cough. Without limiting the “right” sequence exclusively to an expert path, the VP assessment was made to attach different scores to various optional sequences of exploring 2, 3, 4, or 5 symptoms to reflect this type of assessment of diagnostic reasoning.

The fourth domain, interviewing technique, comprises 4 components. The first 3 components, use of a combination of generic vs system-specific questions, transition statements, and number of jumps between topics, are described in Table 1. These 3 components could be easily monitored by the VP. As to the fourth component, colleagues who were consulted for the design of the VP pointed out that specific interviewing pitfalls occurring during medical history taking constituted a key component of their assessment of the performance of students: Did they miss a clue, were they thrown off by a tangential answer, or were they able to stay on course and come back to explore the tangent later? We operationalized these elements through key interview elements (KIEs; see Table 1). These elements, based on the common challenges encountered in interviews, were programmed in a sufficiently large number to ensure that each student would encounter on average 3 or 4 instances.

Each of the 4 domains described earlier were implemented into the VP to provide 4 different scores and a global score: virtual patient–breadth score (VP–BS), virtual patient–depth score (VP–DS), virtual patient–logical sequence score (VP–LSS), and virtual patient–interviewing technique score (VP–ITS), as well as a virtual patient–global score (VP–GS). Although the VP was programmed to provide domain scores from its data, the relative importance of score components and thresholds for specific errors were left to be adapted to the educational context of use.

## Methods

### Design of Study

In this exploratory descriptive study, we articulated and tested a framework for assessing medical history–taking skills with a VP. First, we implemented this framework into a specific VP and then compared global and domain scores assigned by the VP to those assigned by 10 experienced CE participants. The study was approved by our institution’s ethics committee.

### Participants

A total of 10 CEs, all general internists from a Canadian Department of Medicine, were recruited by convenience sampling. The sample consisted of 6 men and 4 women, with a mean (SD) of 16.5 years (9.2) of medical specialty practice and a mean (SD) of 14.3 years (8.3) of evaluating medical students’ history taking. None of the participants had been involved in the elaboration or consultation that had led to the programming of the VP. All participants gave consent to participate in the study.

### Materials

#### Screen Recordings of Student Interviews or Stimuli

Screenshot videos of 3 third-year medical students’ interview with the VP in a clinical case of colon cancer were used as stimuli. Students were recruited through convenience sampling. The screenshot videos were created using Camtasia Studio 7, conserving the students’ anonymity. A total of 2 students were in the first trimester and 1 in the last trimester of the clerkship of a 4-year medical curriculum. Each student was met with individually, and a consent form was signed that authorized the use of recorded data in the research project.

Each student was first introduced to the software. Each part of the screen interface, as well as the navigation boxes, was explained. The student had 10 min to navigate freely and get familiarized with the software. The student was then invited to take a medical history from the VP just as he or she would do with a real patient at the start of a hospital admission. Within a time limit of 30 min, the student was asked to go at his or her own rhythm, without “racing with the clock.” The students readily used the software in all its components, without asking for further explanations. Although the software allowed the students to enter their most likely diagnosis at the end, the screenshot recording was interrupted before they entered their diagnosis, as this was not the focus of the CE’s assessment.

#### Rating Tool

A rating tool was developed for CE by 2 team members (JS and CS). The rating scale mirrored the assessment scheme implemented in the VP with a global performance score and scores for each domain (breadth, depth, logical sequence, and interviewing techniques). Each score was described by 1 question. The CE participants had to provide ratings on descriptive 10-cm visual analog scales with 3 descriptors: 1 at each end labeled “below average” and “above average” and 1 in the middle of the line labeled “average” (see Multimedia Appendix 2), referring in this case to a third-year student’s performance. A visual analog scale was decided upon over a percent score to avoid assessors assigning a typical range of marks between 60% and 100%.

#### Survey on Assessment Practice

A survey was developed by the authors to collect the CE participants’ collective assessment practice. More specifically, the survey documented (a) their relative domain weighting (breadth, depth, logical sequence, interviewing technique) for a global score, (b) their weighting of interviewing technique elements (specific instances, use of statements, use of generic questions, number of jumps between topics), and (c) their acceptable and desirable ranges for the (1) use of statements, (2) use of generic questions, and (3) number of jumps between topics.

To help CE participants better understand some of the terms used (eg, “specific instances”) and how to express the upper and lower limits, the survey included definitions, examples, and visual aids (see Multimedia Appendix 3). 

#### The Virtual Patient Software

The VP has been developed to provide students with feedback on diagnostic reasoning and interviewing skills during medical history taking.

The software entailing three clinical cases including one of colon cancer was developed by the author (JS), a CE who provided the instructional design and content (eg, questions/responses, components of the panels). Instructional design and graphic design support as well as programming in Java were provided by the Instructional Communications Centre, McGill University, Montreal, between 2002 and 2006. The software uses a set of predetermined questions to be used for the interview. Video answers were created to have a set of default responses, provided by an actress, for all the questions available to the medical interviewer. Some reactions of annoyance, irritation, or anxiety were also recorded to keep the interview more realistic. Responses specific to each of the 3 clinical cases were recorded to be substituted to the default questions depending on each clinical situation.

**Figure 1:**
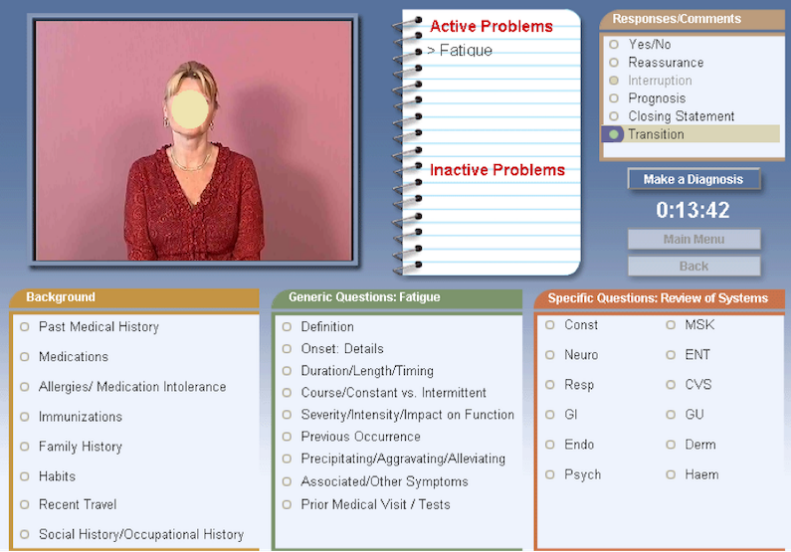
Virtual patient screen interface.

The screen interface consists of various panels (see Figure 1) including the following: (1) a video of the patient, (2) a note pad where the symptoms appear as they are revealed by the patient’s answers that the student can then drag and drop between an active and an inactive problem list, (3) three panels of questions (background questions, generic questions, and a review of systems), (4) a responses-and-comments panel, (5) a clock, (6) a box allowing the student to make the diagnosis at the end, and (7) two buttons (“main menu” and “back”), allowing the student to browse.

The questions available in the software (around 500) are divided into the 3 main categories (illustrated by the different panels shown earlier) and the responses-and-comments panel. The first category (background) includes questions on medical history, medications, allergies, immunizations, family history, habits, recent travel, and social history. The second category lists *generic* questions that can be applied to each symptom, ranging from “What happened just before the symptom started?” to “Have you seen a physician for that symptom?”. Each question can be applied to each of the patient’s symptoms, and the wording of the question changes as the student clicks on a different item on the problem list (on the note pad). The third category consists of a review of systems containing 350 questions. As the student clicks on a system, a list of questions about the chosen system appears. In addition, the student may click on responses or comments that include transition statements, interruption statements, and reassurance statements. A *follow-up* button is available once an answer has been provided by the patient, and allows a choice of 4 follow-up questions: “You need to tell me more about that,” “Let me ask you once more,” “Pardon?,” and “Are you sure?”.

As the patient reveals her symptoms or items of her medical history, they appear in the list of “active problems.” Items can be moved (drag and drop) between the lists of “active problems” and “inactive problems” at any time. When the student is ready, he or she may click on “make a diagnosis” and choose one or more items among a list of diagnoses.

### Procedure

A research assistant met the CE participants for 2 hours. After a brief introduction to the simulation and the project, CE participants had 5 min to navigate for themselves the simulation software, on a different case from the stimulus, to become familiar with the interface and the choice of questions.

The CE participants were then given succinct and nonquantitative definitions of breadth, depth, logical sequence, and interviewing technique, without revealing the corresponding VP operational definitions. After watching each student’s screen recording, they were asked to score the student’s performance using the *rating tool*. They did not see the student’s choice of diagnosis, as the screenshot video was interrupted before, and they were not familiar with the diagnosis of the VP. Afterward, they had to complete the *survey on assessment.*

### Analyses

#### Virtual Patient Scores

The data of the *survey on assessment* were subsequently used to compute the VP-derived scores for these components. For example, to compute the VP–GS, each VP domain score (breadth, depth, logical sequence, and interviewing technique) was multiplied by the mean weight that the CE participants attached to each domain. Furthermore, to compute the VP–ITS, the CE participants’ mean suggested cutoffs were used for acceptable and optimal ranges of generic vs specific questions, transitioning statements, and number of jumps between topics.

#### Clinician-Educator Scores

The response on each participant’s visual analog scale was converted to a score out of 100 by measuring with a ruler the position of the respondent’s pen mark, with 10 cm representing 100%. For each student, the mean values for global performance, breadth, depth, logical sequence, and interviewing technique scores provided by the 10 CE participants on the assessment grids constituted the CE scores (clinician-educator–global score [CE–GS], clinician-educator–breadth score [CE–BS], clinician-educator–depth score [CE–DS], clinician-educator–logical sequence score [CE–LSS], clinician-educator–interviewing technique score [CE–ITS], respectively).

## Results

**Figure 2:**
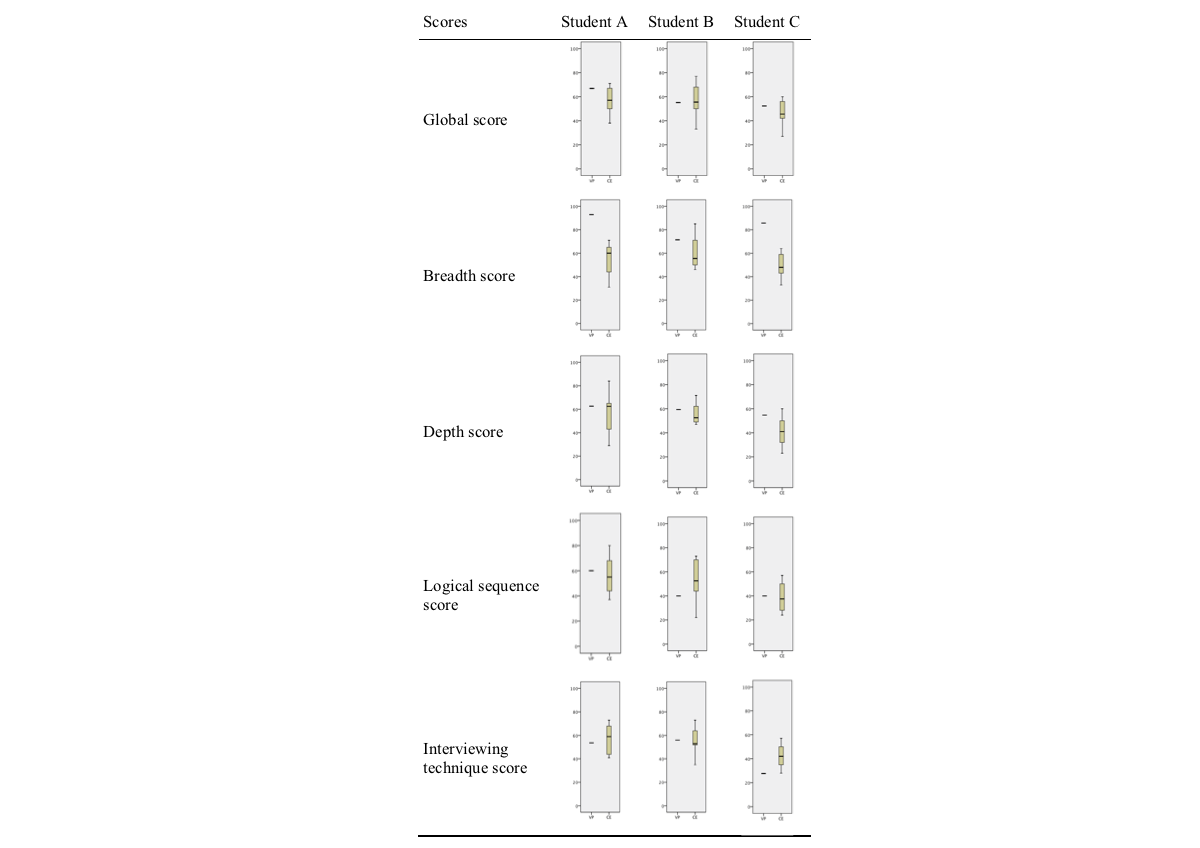
Boxplots displaying the virtual patient and clinician-educator scores for each student and for each score.

Students’ scores, from VP and CE, are presented in Figure 2. The single line represents the VP software assessment, and the boxplot represents the range of assessment made by the CE participants. There is a boxplot for each of the 3 students’ performance for each of the 5 scores. The goal of these descriptive analyses is to explore how the assessment provided by the VP using our framework compares with the gold standard, that is, the assessment provided by the CE. The aim is having a VP score that is within the range of scores that CE have assigned to each student (see Multimedia Appendix 4).

Overall, the scores provided by the VP were slightly higher but comparable with the ones assigned by the CE for the global performance and for the domains of depth, logical sequence, and interviewing technique. For breadth, the VP scores were higher, and they did not fall within the range of the CE scores for student A and C. On interviewing technique, which includes 4 components, only the score for student C from the VP was not within the range of CE scores.

## Discussion

### Comparing Virtual Patient and Clinician-Educators’ Scores

We implemented within a specific VP a framework for assessing medical history taking by breaking down broad skills into bite-sized assessment points and then tested the framework against the judgment of 10 CEs. Our findings suggest that through such a framework, assessment by VP can produce scores akin to those generated by a CE. We discuss our results and reflect on the relevance of each domain in terms of the proposed assessment scheme and on its implementation.

An advantage of using an assessment framework embedded in a VP to assess medical history–taking skills is the reliability of the assessment [34]. When referring to reliability as “the consistency of scores across replications of a testing procedure” [34], it is clear that automated assessment can contribute to the reliability of the assessment. Given that reliability is often considered necessary to the validity of the assessment scores, it stands to reason that we wanted to document if our framework embedded in a VP yielded valid assessments. Thus, we compared VP scores with “gold standards,” that is, CEs’ assessment of the history-taking skills of medical students.

The VP–GS was computed from the 4 domain scores, albeit with relative weighting calibrated according to the survey of CE participants (see Multimedia Appendix 4 for details).The CE–GSs were an appraisal by CE of the students’ overall performance and were not derived from the CE’s domain scores. CE’s global appraisals typically have the gestalt quality of a true expert assessment [35] and represent the gold standard for the VP–GS. Overall, the global scores from the VP fell within the range of the CE–GSs, suggesting that depth, breadth, logical sequence, and interviewing technique are appropriate and sufficient domains to approximate an expert’s gestalt assessment, as otherwise VP– and CE–GSs might have differed. Furthermore, when CEs were asked for additional domains that they considered important, they named aspects of the medical interview that could not be seen on a screen recording, such as students’ empathy, body language, and tone; skills such as picking up nonverbal clues; and the ability to organize the interview between introduction and conclusion, which they felt, rightly, that the VP did not allow. It bears to be pointed out that some of these aspects of assessing medical history taking fall outside the “gathering information” section of the Calgary-Cambridge model, whereas others such as picking up nonverbal clues are within that section and could have been programmed into a VP (eg, video of the VP fidgeting) but were not addressed by our framework.

The VP scores for breadth, that is, the identification of the full range of the VP’s various symptoms, are higher than the breadth scores given by CE for 2 of the 3 students. VP scores were simply the percentage of symptoms identified by the student out of the total number of symptoms programmed. Unlike the VP, CE had no knowledge of the total number of symptoms programmed and made a judgment as to what other symptoms this type of patient might have. There could be two main reasons for this difference between VP– and CE–BSs: (1) the VP may not have been programmed with a sufficiently large number of symptoms to be a realistic representation of this type of patient or (2) CE may have expected a broader range of questions about general symptoms, the so-called “review of systems.” We did not identify missing details that should have been programmed into the VP after repeated use of the case with students and consultation with CE, suggesting that rather than the VP having too few symptoms, CEs expect a review of systems as part of any medical history taking. Of note, all 10 CE participants were general internists, who likely incorporate such a generalist approach in their own practice. The review of systems was not taken into account in the VP–BS.

The VP scores for depth, that is, the level of detail about each of the VP’s symptoms, are within the range of scores given by CEs. Again, VP scores were simply the percentage of symptom details identified by the student out of the total number of symptom details programmed. The fact that the CE’s judgment is aligned with this simple ratio suggests that CEs were able to estimate the details about symptoms that were missed or not missed by the students.

The VP scores for logical sequence, which reflects systematic thinking through the relevant diagnostic possibilities, are well aligned with the range of scores given by the CE. Implementation in a VP was much more complex than that for breadth or depth as it involved assigning different scores to a number of potential sequences of questions relevant to the VP’s symptoms. Indeed, this domain required a set of rules that reflected the existence, as for all complex problem solving, of not just one so-called expert path, but of several acceptable paths to reaching the diagnosis. In addition, this domain score, unlike the first 2, could not be improved by the students simply clicking on as many questions as they could, as the scoring depended on sequence of questioning rather than the sheer number of questions asked.

The VP scores for interviewing technique, which is a combination of 4 components (appropriate use of generic questions, transition, flow, and handling of KIEs), are within the ranges of scores by CE for students A and B and slightly less for student C. This other complex measure, which has been calibrated using the ranges suggested by CEs as to the ideal and acceptable limits for the number of jumps between topics, the use of transitioning statements, and the use of generic questions and specific KIEs, seems to provide VP scores that are in the lower range than the corresponding CE scores. The VP scores were binary and may have been too restrictive in their application of CE’s suggested ideal and acceptable ranges.

### Survey on Assessment Practice

The responses of the 10 CEs to the study survey documented their relative domain weighting (breadth, depth, logical sequence, and interviewing technique) for a global score, their weighting of interviewing technique elements (specific instances, use of statements, use of generic questions, and the number of jumps between topics), and their acceptable and desirable ranges for the use of statements, the use of generic questions, and the number of jumps between topics (see Multimedia Appendix 4). These surveys allowed us to refine the framework at the final step of computing scores from the raw data of the VP. Such an iterative process ensured that an automated assessment reflected the CE’s priorities and values in judging student performance.

### Reflection on Proposed Outcome Measures

Developing a framework for assessment of history-taking skills to program into a VP and comparing VP scores with CEs’ judgment enables us to reflect both on the proposed framework and on its implementation into a specific VP. For example, as we reflect on how the breadth score is underestimated by the VP, we know we are probably missing an element of breadth as defined by CEs, likely a wider-ranging review of systems, as described earlier. We are therefore considering the integration of an additional component of the number of systems (eg, cardiovascular, renal) the student explores through specific questions into the VP’s domain score. Similarly, when we are reflecting on the implementation of our framework, we want to review how the ranges of acceptable numbers of generic questions or transition statements are calculated. Instead of applying discrete cutoffs (eg, less than 26.4% is given zero, based on the mean from the CE survey), we would possibly need to try using an incremental cutoff to better reflect CEs’ judgment and resulting scores.

Numerous studies related to VPs have centered on their impact on knowledge acquisition and skills [36]. This study focuses on developing an assessment framework aligned with educators’ assessment practices. Inviting CEs’ perspective [1,37] allows for the creation of VP aligned with CEs’ educational objectives, while in turn providing CE with an opportunity to understand better their students’ skill development. After implementation, using CEs’ judgment to validate and test the assessment framework, as we have done here, further helps improve implementation and alignment with objectives. The ultimate goal is better VP integration into the formal curriculum, and a smooth transition from VP to bedside teaching, as it is clear that no VP could ever replace real interaction with patients. Assessment provided by VP must make sense to all actors in the learning environment, and reflect as faithfully as possible current assessment practices, ultimately to promote genuine improvement in performance.

### Limitations

The study’s CE vs VP comparison results are preliminary, as they include the use of a single case and limited number of students’ performances. Our results need to be tested with other cases and a larger audience in a variety of settings. Medical students at the clerkship level are the intended audience for this specific VP software dealing with diagnostic reasoning and interviewing skills, and the results may not hold true for different levels of students and additional assessments such as communication skills and body language. In addition to the small number of students’ performances, their narrow spread represents another limitation. The 3 students did not have extremes of high- and low-quality performance. Using a larger pool of students and selecting specific performances purposefully for validating a broad range of performance would enable us to test better for VP scores’ discriminative ability. Also, this VP software is not intended to assess the nonverbal communication skills inherent to the history-taking skills, the focus being more on most of the other aspects of gathering information as part of the medical interview.

### Conclusions

We developed a framework for assessment of medical history–taking skills and programmed it into a VP software that aligned with assessment by CEs in our small observational study. Through an iterative process, our study also provided insight into how CEs assess specific domains of medical history taking, allowing us to refine further the scheme programmed into the VP. Our results suggest that some skills that are usually assessed at the bedside can be assessed by software, provided reasoning is judged with flexibility through a range of logical sequences rather than an “expert path” and that broad descriptive terms such as “picks up clues” can be translated into operational, observable behaviors by the student and the VP is then specifically programmed to include situations that call upon the student to demonstrate these skills by engaging in specific behaviors (such as clarification, following up on clues, asking a logical sequence of questions, using open-ended questions) Further steps in this direction, with more diverse VPs and ongoing consultation and exchange with CEs can be expected to result in producing a generation of VPs that are programmed to provide feedback to learners and to assist teachers in their assessment of performance.

## Appendix

Multimedia Appendix 1 Differential scoring for overall order of identification of symptoms and for alternative sequences.

Multimedia Appendix 2 Rating tool.

Multimedia Appendix 3 Survey on assessment practice.

Multimedia Appendix 4 Mean weight for each domain and components and mean limits for acceptable and optimal range for domains and components given by clinician-educator participants.

## Abbreviations

BIC: Brown Interviewing Checklist
CE: clinician-educator
CE–BS: clinician-educator–breadth score
CE–DS: clinician-educator–depth score
CE–GS: clinician-educator–global score
CE–ITS: clinician-educator–interviewing technique score
CE–LSS: clinician-educator–logical sequence score
HPE: Health Professions Education
HTRS: History-Taking Rating Scale
KIE: key interview element
MAAS: Maastricht History-taking and Advice Checklist
VP: virtual patient
VP–BS: virtual patient–breadth score
VP–DS: virtual patient–depth score
VP–GS: virtual patient–global score
VP–ITS: virtual patient–interviewing technique score
VP–LSS: virtual patient–logical sequence score
