# Supplementary material for: Comparison of Assessment by a Virtual Patient and by Clinician-Educators of Medical Students' History-Taking Skills: Exploratory Descriptive Study
Source: JMIR Med Educ. 2020 Mar 12;6(1):e14428. doi: 10.2196/14428 (PMC7099396; doi:10.2196/14428)
Supplement: Multimedia Appendix 1 [file mededu_v6i1e14428_app1.pdf]

Multimedia Appendix 1

Differential scoring for overall order of identification of symptoms; and for alternative sequences

EXPERT PATHS	CONDITIONS	SCORE (maximum=100/100)
Fatigue; dizziness; pallor; blood in the stools; constipation	Sequence obtained	100
Fatigue; pallor; dizziness; blood in the stools; constipation		80
Fatigue; pallor; blood in the stools; constipation		80
Fatigue; pallor; blood in the stools; dizziness; constipation		80
Fatigue; pallor; dizziness; constipation; blood in the stools		40
Fatigue; dizziness; pallor; constipation; blood in the stools		40
Fatigue; dizziness	Consecutive sequence: no intercalations	20
Fatigue; pallor		20
Pallor; blood in the Stools		20
Blood in the stools; constipation		20
Dizziness; pallor		20
Blood in the Stools; pallor		20
Constipation; blood in the stools		20
Pallor; dizziness		20
