# Supplementary material for: Comparison of Assessment by a Virtual Patient and by Clinician-Educators of Medical Students' History-Taking Skills: Exploratory Descriptive Study
Source: JMIR Med Educ. 2020 Mar 12;6(1):e14428. doi: 10.2196/14428 (PMC7099396; doi:10.2196/14428)
Supplement: Multimedia Appendix 2 [file mededu_v6i1e14428_app2.pdf]

Multimedia Appendix 2

Rating tool

Assessment of Student's Performance

For each of the statements below, please indicate by placing a mark on the line, your evaluation of the student's performance (see visual aid 1).

1. Student's Global Performance

Average

Below average | Above average

2. Breadth: the student explored all the relevant problems.

Average

Below average | Above average

3. Depth: the student explored the problems in sufficient detail.

Average

Below average | Above average

4. Logical sequence: the student followed a logical sequence denoting sound clinical reasoning to obtain the relevant information.

Average

Below average | Above average

5. Interviewing skills: the student performed a good quality interview.

Average

Below average | Above average
