# Supplementary material for: Comparison of Assessment by a Virtual Patient and by Clinician-Educators of Medical Students' History-Taking Skills: Exploratory Descriptive Study
Source: JMIR Med Educ. 2020 Mar 12;6(1):e14428. doi: 10.2196/14428 (PMC7099396; doi:10.2196/14428)
Supplement: Multimedia Appendix 3 [file mededu_v6i1e14428_app3.pdf]

Multimedia Appendix 3

Assessment of a student's medical interview

1- Indicate, for the assessment of a student's medical interview with a patient, the relative weight you usually assign to each of the following domains (for a total of 100):

Domains	Percentage
Breadth of data	%
Depth of data	%
Logical sequence	%
Interviewing technique	%
TOTAL	100 %

2- What other components of an interview do you take into account for the assessment of a student's medical interview with a patient?

Interviewing Technique

3- When assessing student's interviewing skills, what is, for you, the relative weight (in percentages) that should be given to each of the following components (for a total of 100%):

Components of Interviewing Technique	Definition	Relative weight
Successful handling of key interview elements (KIE)	Situations used to assess students' interviewing skills has been integrated in this simulated interview. An interview like the one you just watched contained about ten KIE	%
Appropriate use of transitioning statements	Transitioning sentence /reassuring sentences /yes/no (as opposed to a question) (upper-right panel).	%
Appropriate use of generic questions	Questions that comes from the middle panel	%
Number of jumps between topics	Jumps between topics leaving some areas not fully explored.	%
TOTAL		100%

Transitioning statements, appropriate use of generic questions, and jumps between topics

Often, novice students use too few or too many transitioning statements or generic questions. For both of these components, please indicate in percentages: the lower acceptable limit, the lower desirable limit, the upper desirable limit, and the upper acceptable limit.

4- Percentage of transitioning statements (see visual aid 1)

- The lower limit of acceptable % of transitioning statements: ______%
- The lower limit of desirable % of transitioning statements: ______%
- The upper limit of desirable % of transitioning statements: ______%
- The upper limit of acceptable % of transitioning statements: ______%

5- Percentage of generic questions (see visual aid 2)

- The lower limit of acceptable % of generic questions: ______%
- The lower limit of desirable % of generic questions: ______%
- The upper limit of desirable % of generic questions: ______%
- The upper limit of acceptable % of generic questions: ______%

6- Number of jumps between topics (see visual aid 3)

Novice students may have too many jumps between topics, leaving some areas not fully explored during a medical interview. Please indicate:

- The upper limit of acceptable number of jumps between topics: ______
- The upper limit of desirable number of jumps between topics: ______
