# Supplementary material for: Comparison of Assessment by a Virtual Patient and by Clinician-Educators of Medical Students' History-Taking Skills: Exploratory Descriptive Study
Source: JMIR Med Educ. 2020 Mar 12;6(1):e14428. doi: 10.2196/14428 (PMC7099396; doi:10.2196/14428)
Supplement: Multimedia Appendix 4 [file mededu_v6i1e14428_app4.pdf]

Multimedia Appendix 4

Mean weight for each domain and components, and mean limits for acceptable and optimal range for domains and components given by CE participants

Domains	Mean weighting of domains from survey of CE participants (%)	Mean weighting of components from survey of CE participants (%)	Mean limits from survey of CE participants
Breadth	22.0		Not applicable
Depth	29.0		Not applicable
Logical sequence	32.5		Not applicable
Interview technique	16.5		Not applicable
Component			
a) Appropriate use of generic questions		14.0	(%)<br>Acceptable: 26.0- 62.5<br>Optimal: 33.0 - 52.0
b) Appropriate use of transitioning statements		30.0	(%)<br>Acceptable: 5.95- 22.50<br>Optimal:<br>10.55 -16.25
c) Appropriate flow		36.0	(number of jumps among topics)<br>Optimal: ≤ 4,30<br>Acceptable: ≤ 8.40
d) Successful handling of key interview elements (KIE)		20.0	na
